# Regulation of Vascular Endothelial Cell Polarization and Migration by Hsp70/Hsp90-Organizing Protein

**DOI:** 10.1371/journal.pone.0036389

**Published:** 2012-04-30

**Authors:** Jingyu Li, Xiaodong Sun, Zaizhu Wang, Li Chen, Dengwen Li, Jun Zhou, Min Liu

**Affiliations:** 1 State Key Laboratory of Medicinal Chemical Biology, College of Life Sciences, Nankai University, Tianjin, China; 2 Key Laboratory of Immune Microenvironment and Disease of the Ministry of Education, Basic Medical College, Tianjin Medical University, Tianjin, China; Bristol Heart Institute, University of Bristol, United Kingdom

## Abstract

Hsp70/Hsp90-organizing protein (HOP) is a member of the co-chaperone family, which directly binds to chaperones to regulate their activities. The participation of HOP in cell motility and endothelial cell functions remains largely unknown. In this study, we demonstrate that HOP is critically involved in endothelial cell migration and angiogenesis. Tube formation and capillary sprouting experiments reveal that depletion of HOP expression significantly inhibits vessel formation from endothelial cells. Wound healing and transwell migration assays show that HOP is important for endothelial cell migration. By examination of centrosome reorientation and membrane ruffle dynamics, we find that HOP plays a crucial role in the establishment of cell polarity in response to migratory stimulus. Furthermore, our data show that HOP interacts with tubulin and colocalizes with microtubules in endothelial cells. These findings indicate HOP as a novel regulator of angiogenesis that functions through promoting vascular endothelial cell polarization and migration.

## Introduction

Hsp70/Hsp90-organizing protein (HOP) is a co-chaperone protein that could directly bind to the major chaperones, Hsp70 and Hsp90, and modulate their ATPase activities [1]–[4]. HOP also belongs to the stress-inducible protein 1 (STI1) family, which contains various vertebrate and invertebrate homologs of HOP [5]. Human HOP has three tetratricopeptide repeat (TPR) domains, including TPR1, TPR2a, and TPR2b [6]. TPR domains are responsible for the interaction of HOP with Hsp70/Hsp90, with TPR1 for HOP binding to Hsp70 and TPR2a for HOP binding to Hsp90 [7], [8]. However, no specific ligands for the TPR2b domain have been found. HOP also has non-TPR domains, including a DP repeat region and two nuclear localization signals, which render HOP to dynamically translocate between the cytoplasm and the nucleus [9], [10]. In addition, HOP has been found in a number of Hsp90-independent complexes, including prion protein complex and nuclear transcription complex [11]–[14].

Hsp70 and Hsp90 have been shown to be overexpressed in several tumor types, and inhibition of Hsp70/Hsp90 has been reported to induce tumor cell-specific apoptosis [15]. However, whether HOP is involved in cancer development remains unclear. In addition, it is unknown whether HOP plays a role in angiogenesis,a process that refers to the generation of new blood vessels from pre-existing ones and relies largely on vascular endothelial cell proliferation and migration [16]. The angiogenic process is also critical for tumor growth, progression, and metastasis [17], [18], and anti-angiogenesis strategy has been proven useful for cancer treatment [19]–[22]. Given that HOP harbors multiple structural motifs and interacts with various proteins, we hypothesized that HOP might participate in cell motility and endothelial cell functions. This study was designed to test this hypothesis directly, and our data demonstrate that HOP is important for angiogenesis through modulating endothelial cell polarization and migration.

## Materials and Methods

### Materials

Antibodies against β-actin, α-tubulin, γ-tubulin, and GST were purchased from Sigma-Aldrich. Antibodies against GFP, RhoA, Cdc42, and Rac1 were from Cell Signaling. Antibodies against HOP, Akt, and HDAC6 were from Santa Cruz Biotechnology. Anti-GFP antibody-conjugated agarose beads (MBL International), glutathione-conjugated agarose beads (Sigma-Aldrich), horseradish peroxidase-conjugated secondary antibodies (Amersham Biosciences), and fluorescein- or rhodamine-conjugated secondary antibodies (Jackson Immuno Research Laboratories) were obtained from the indicated sources. Matrigel and mouse collagen IV were from BD Biosciences.

### Plasmids

Mammalian expression plasmids of GFP-HOP and GFP-tagged mutant forms of HOP were constructed using the pEGFPC1 vector. Bacterial expression plasmid of GST-HOP was cloned using the pGEX6P3 vector, and GST-HOP fusion protein was purified by glutathione-Sepharose 4B beads according to the manufacturer's instructions (Promega).

### Cell Culture and Transfection

Human umbilical vein endothelial cells (HUVECs, #CRL-1730), HeLa (#CCL-2), and 293T (#CRL-11268) cells were obtained from the American Type Culture Collection. The use of HUVECs, HeLa, and 293T cells was approved by the Ethics Committee of Nankai University. HeLa and 293T cells in Dulbecco's modified Eagle's medium and HUVECs in RPMI 1640 medium were cultured in the indicated media supplemented with 10% fetal bovine serum at 37°C in a humidified atmosphere with 5% CO_2_. Plasmids were transfected into HeLa and 293T cells using polyethylenimine and transfected into HUVECs by electroporation with the ECM830 system (BTX). siRNAs were synthesized by Dharmacon and transfected with the RNAiMAX reagent (Invitrogen).

### In Vitro Angiogenesis Assays

To examine the formation of tube-like structures, HUVECs were seeded onto 6-well plates precoated with matrigel, and tube formation was then examined at different time points. The extent of tube formation was quantified by measuring the cumulative tube length with ImageJ (National Institutes of Health). To examine capillary sprouting, HUVECs were suspended in culture medium containing 0.25% carboxymethylcellulose and seeded onto round-bottom 96-well plates to form spheroids. The spheroids were then embedded into collagen gels in 24-well plates, and culture medium was added on top of the gel. Capillary-like sprout formation was then examined by microscopy and angiogenic activity was quantified by measuring the cumulative sprout length per spheroid.

### Cell Migration Assays

To examine wound healing, HUVECs grown in 24-well plates as conﬂuent monolayers were mechanically scratched using a pipette tip to create the wound. Cells were washed with phosphate-buffered saline to remove the debris, and complete culture medium was added to allow for wound healing. Phase contrast images of the wound were then taken at different time points as described previously [23]. To analyze the transwell migratory activity of HUVECs, the upper surface of the transwell filters was coated with matrigel. Cells suspended in 200 µl serum-free media were then added to the chamber, and the chamber was placed in a 24-well plate containing complete medium. After 24-hour incubation at 37°C, the filters were gently taken out and matrigel on the upper surface of the filters was removed by cotton swabs. Cells on the underside of transwell filters were fixed with 4% paraformaldehyde for 30 minutes, stained with 0.1% crystal violet for 10 minutes, and then photographed. The extent of cell migration was quantified as the number of migrated cells in the drug-treatment group divided by the number of migrated cells in the control group as described [24].

### Cell Proliferation and Apoptosis Assays

Sulforhodamine B staining assay was used to examine cell proliferation. In brief, cells were stained with 0.4% sulforhodamine B (Sigma-Aldrich) dissolved in 1% acetic acid. The cells were then washed with 1% acetic acid to remove unbound dye. The protein-bound dye was extracted with 10 mM Tris base to determine the optical density at 490-nm wavelength as described previously [25]–[27]. Annexin V staining assay was performed to analyze the apoptotic rate. Cells were stained with fluorescein-conjugated annexin V (Invitrogen), and phase contrast and fluorescence images were then taken with an Axio Observer A1 fluorescence microscope (Carl Zeiss) as described previously [28].

### Examination of Cell Polarization

HUVECs transfected with HOP or control siRNAs were scratched, and cells were fixed 2 hours later and stained with anti-α-tubulin antibody, anti-γ-tubulin antibody, and DAPI to visualize microtubules (green), centrosomes (red), and nuclei (blue), respectively. To analyze membrane ruffle dynamics at the leading edge of migrating cells, cells transfected with the pEGFPC1 vector were cultured in a 37°C chamber on a TCS SP5 confocal microscope (Leica), equipped with a live-cell imaging workstation. The ﬂuorescence of GFP at the leading edge of cells was recorded at 20-second intervals using the LASAF software. The acquired image sequences were analyzed by ImageJ and membrane rufﬂe dynamics were presented as three-dimensional surface plots.

### Immunofluorescence Microscopy

Cells grown on glass coverslips were fixed with 4% paraformaldehyde for 30 minutes at room temperature and blocked with 2% bovine serine albumin in phosphate-buffered saline. Cells were incubated with primary antibodies and then with fluorescein or rhodamine conjugated secondary antibodies followed by staining with DAPI as described previously [29]–[31]. Coverslips were then examined with an Axio Observer A1 fluorescence microscope (Carl Zeiss).

### Immuoprecipitation and GST-Pulldown

For immunoprecipitation, cell lysate was incubated with anti-GFP antibody-conjugated agarose at 4°C for 2 hours, and the precipitated proteins were then examined by immunoblotting. For GST-pulldown, cell lysate was incubated with bacterially purified GST or GST-HOP proteins immobilized on glutathione-conjugated agarose at 4°C for 2 hours. The pulldown preparations were then analyzed by immunoblotting.

### Immunoblotting

Cells were lysed in a buffer containing 1% Triton X-100, 150 mM NaCl, and 50 mM Tris (pH 7.5). Proteins were resolved by SDS-PAGE and transferred onto polyvinylidene difluoride membranes (Millipore). The membranes were blocked in Tris-buffered saline containing 0.2% Tween 20 and 5% fat-free dry milk and incubated with primary antibody and then with horseradish peroxidase-conjugated secondary antibody as described [32], [33]. Specific proteins were visualized with enhanced chemiluminescence detection reagent (Millipore).

## Results

### HOP Promotes Endothelial Tube Formation and Sprouting

**Figure 1:**
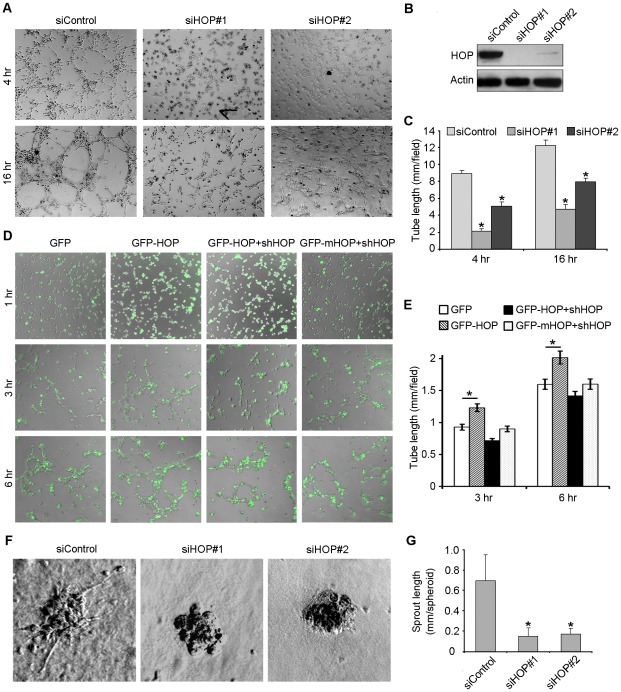
HOP promotes endothelial tube formation and sprouting. **A.** HUVECs transfected with HOP or control siRNAs were plated onto matrigel, and photographs were taken 4 and 16 hours later. **B.** Immunoblotting analysis of HOP and β-actin expression in HUVECs transfected with HOP or control siRNAs. **C.** Experiments were performed as in A, and the cumulative tube length was measured. *, p<0.05 versus control. **D.** HUVECs transfected with GFP, GFP-HOP, GFP-HOP plus HOP shRNA, or GFP-mHOP (shRNA-resistant mutant) plus HOP shRNA were plated onto matrigel, and photographs were taken 1, 3, and 6 hours later. **E.** Experiments were performed as in D, and the cumulative tube length was measured. *, p<0.05. **F.** Capillary-like sprout formation from spheroids generated from HUVECs transfected with HOP or control siRNAs. **G.** Experiments were performed as in F, and the cumulative sprout length was measured. *, p<0.05 versus control.

To investigate the possible involvement of HOP in angiogenesis, we performed tube formation assay. HUVECs were transfected with HOP or control siRNAs and plated onto matrigel. Four hours later, cells transfected with control siRNA could form tube-like structures and 16 hours later, the tube-like structures became more significant. In contrast, knockdown of HOP expression apparently inhibited tube formation (Figure 1A–1C). We also found that overexpression of HOP could enhance the ability of HUVECs to form tubes (Figure 1D and 1E). In addition, we found that HOP shRNA could abolish the action of wild-type HOP in endothelial tube formation, but not the action of shRNA-resistant mutant HOP in tube formation (Figure 1D and 1E), validating the specificity of the interference experiments.

We then performed capillary sprouting assay to further examine the role of HOP in angiogenesis. We found that the ability of endothelial cell spheroids to form capillary-like sprouts was significantly impaired by transfection with HOP siRNAs (Figure 1F). Calculation of sprout length showed that the #1 and #2 HOP siRNAs decreased sprout length by 71% and 74%, respectively (Figure 1G). These data indicate that HOP is important for angiogenesis in vitro.

### Knockdown of HOP Expression Does Not Affect the Proliferative Behavior or Apoptotic Rate of Vascular Endothelial Cells

**Figure 2:**
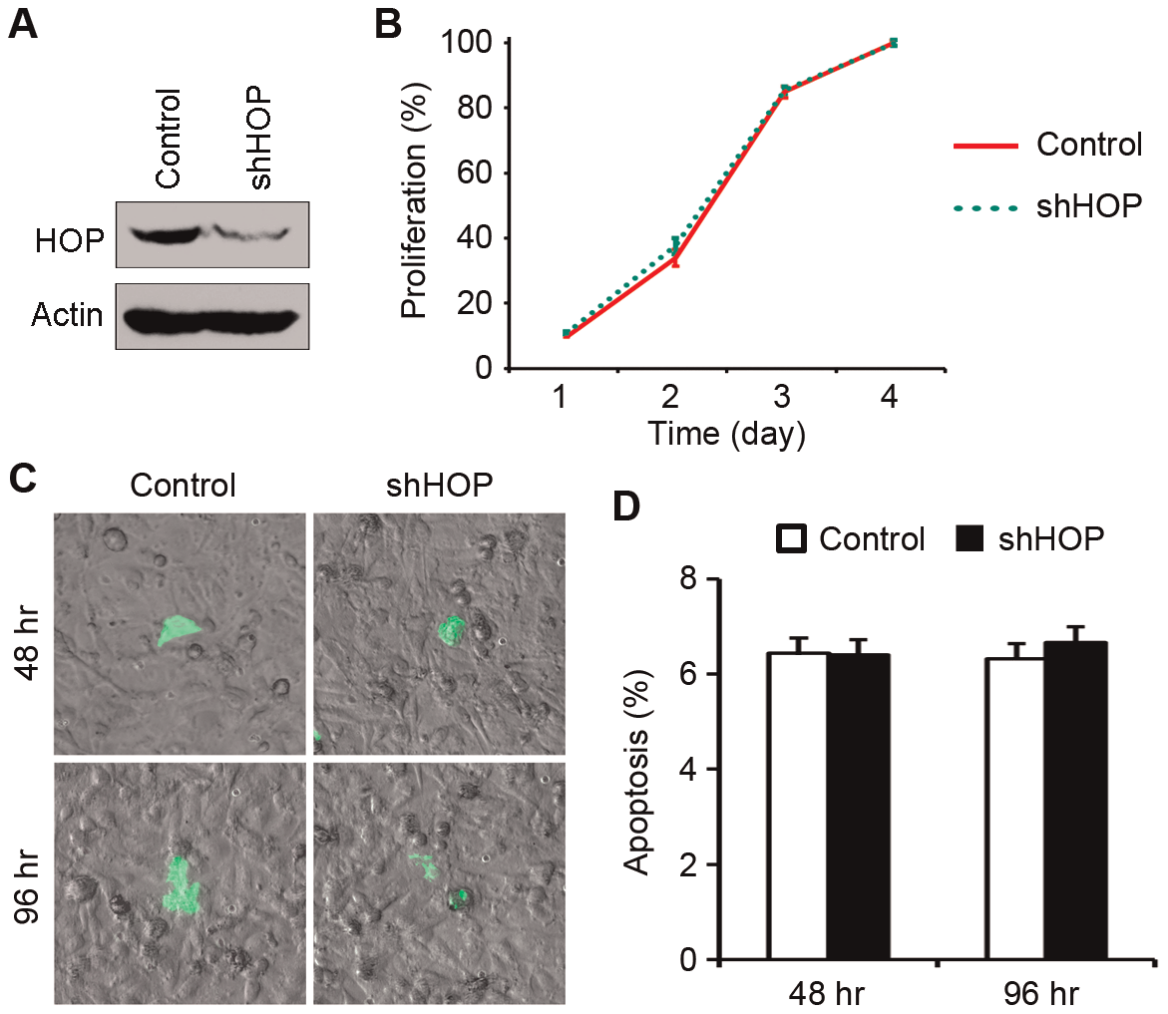
Knockdown of HOP expression does not affect the proliferative behavior or apoptotic rate of vascular endothelial cells. **A.** Immunoblotting analysis of HOP and β-actin expression in HUVECs transfected with HOP shRNA or the control vector for 96 hours. **B.** Growth curves of HUVECs transfected with HOP shRNA or the control vector, measured by sulforhodamine B staining assay. **C.** HUVECs transfected with HOP shRNA or the control vector for 48 or 96 hours were stained with fluorescein-conjugated annexin V. Phase contrast and fluorescence images were then taken. **D.** Experiments were performed as in C, and the percentage of apoptotic cells was then quantified.

We next investigated the molecular mechanism of how HOP is involved in angiogenesis. The angiogenic process requires both the proliferation and migration of vascular endothelial cells [16]. We thus examined whether the depletion of HOP in HUVECs affects their proliferative behavior. HOP shRNA could significantly inhibit the expression of HOP (Figure 2A), but cells transfected with HOP shRNA or the control vector showed very similar growth curves (Figure 2B). This result suggests that knockdown of HOP expression does not affect vascular endothelial cell proliferation.

Heat shock proteins have been also involved in apoptosis resistance. We thus investigated the effect of HOP depletion on the apoptotic rate of vascular endothelial cells. HUVECs transfected with HOP shRNA or the control vector were stained with fluorescein-conjugated annexin V, which reports the loss of phosphatidylserine asymmetry of the plasma membrane at the early stage of apoptosis [34]. We found that the depletion of HOP does not alter the apoptotic rate of vascular endothelial cells (Figure 2C and 2D).

### HOP Is Important for Endothelial Cell Migration

**Figure 3:**
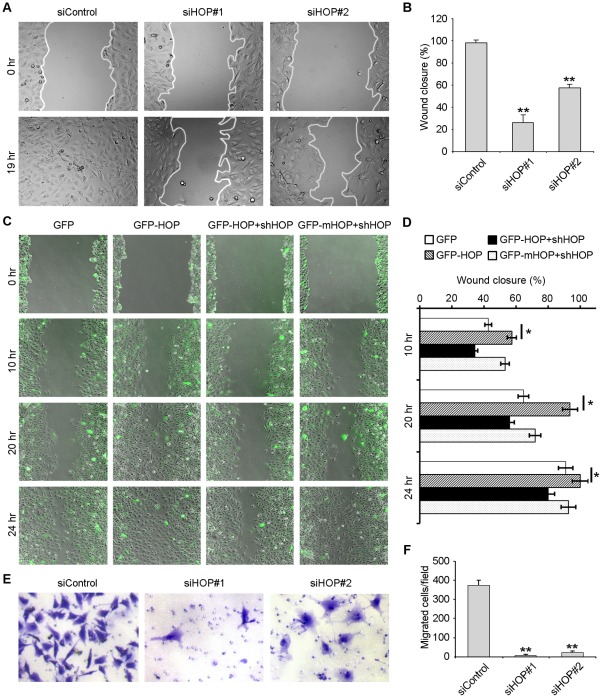
HOP is important for endothelial cell migration. **A.** HUVECs transfected with HOP or control siRNAs were scratched, and wound margins were imaged 0 or 19 hours later. **B.** Experiments were performed as in A, and the extent of wound closure was quantified by measuring the wound area compared with the initial wound area. **, p<0.01 versus control. **C.** HUVECs transfected with GFP, GFP-HOP, GFP-HOP plus HOP shRNA, or GFP-mHOP plus HOP shRNA were scratched, and wound margins were imaged 0, 10, 20, or 24 hours later. **D.** Experiments were performed as in C, and the extent of wound closure was quantified. *, p<0.05. **E.** HUVECs transfected with HOP or control siRNAs for 84 hours were placed in transwell migration chambers containing filters coated uniformly on upper side with matrigel. To stimulate migration, 10% FBS was added to the lower chamber. Transwell chambers were stained with crystal violet and imaged after 12 hours. **F.** Experiments were performed as in E, and the average number of migrated cells was quantified. **, p<0.01 versus control.

Migration of endothelial cells is an important step of angiogenesis [35], [36]. Therefore, we next wanted to find out whether HOP functions in angiogenesis through regulating endothelial cell migration. HUVECs transfected with HOP or control siRNAs were mechanically scratched to create a wound. After 19 hours, we examined the extent of wound closure. By measuring the wound area compared with the initial wound area, we found that the ability of HUVECs to migrate to the wound area was significantly inhibited after transfection with HOP siRNAs (Figure 3A and 3B). We further found that overexpression of HOP promoted the migration of HUVECs (Figure 3C and 3D). In addition, HOP shRNA inhibited the effect of wild-type HOP on cell migration, but not the effect of shRNA-resistant mutant HOP on cell migration (Figure 3C and 3D).

To verify the role of HOP in cell migration, we performed a transwell migration assay, which was routinely used to study cell migration in response to specific signal stimuli. HUVECs transfected with HOP or control siRNAs were placed in transwell migration chambers containing filters coated with matrigel on the upper side. We examined the number of cells that migrate through matrigel in response to serum stimulation. As shown in Figure 3E and 3F, only a small population of cells transfected with HOP siRNAs could pass through the matrigel, whereas cells transfected with the control siRNA migrated efficiently. These results thus suggest that HOP regulates angiogenesis through promoting endothelial cell migration.

### HOP Regulates Cell Polarization and Membrane Ruffling in Migrating Cells

**Figure 4:**
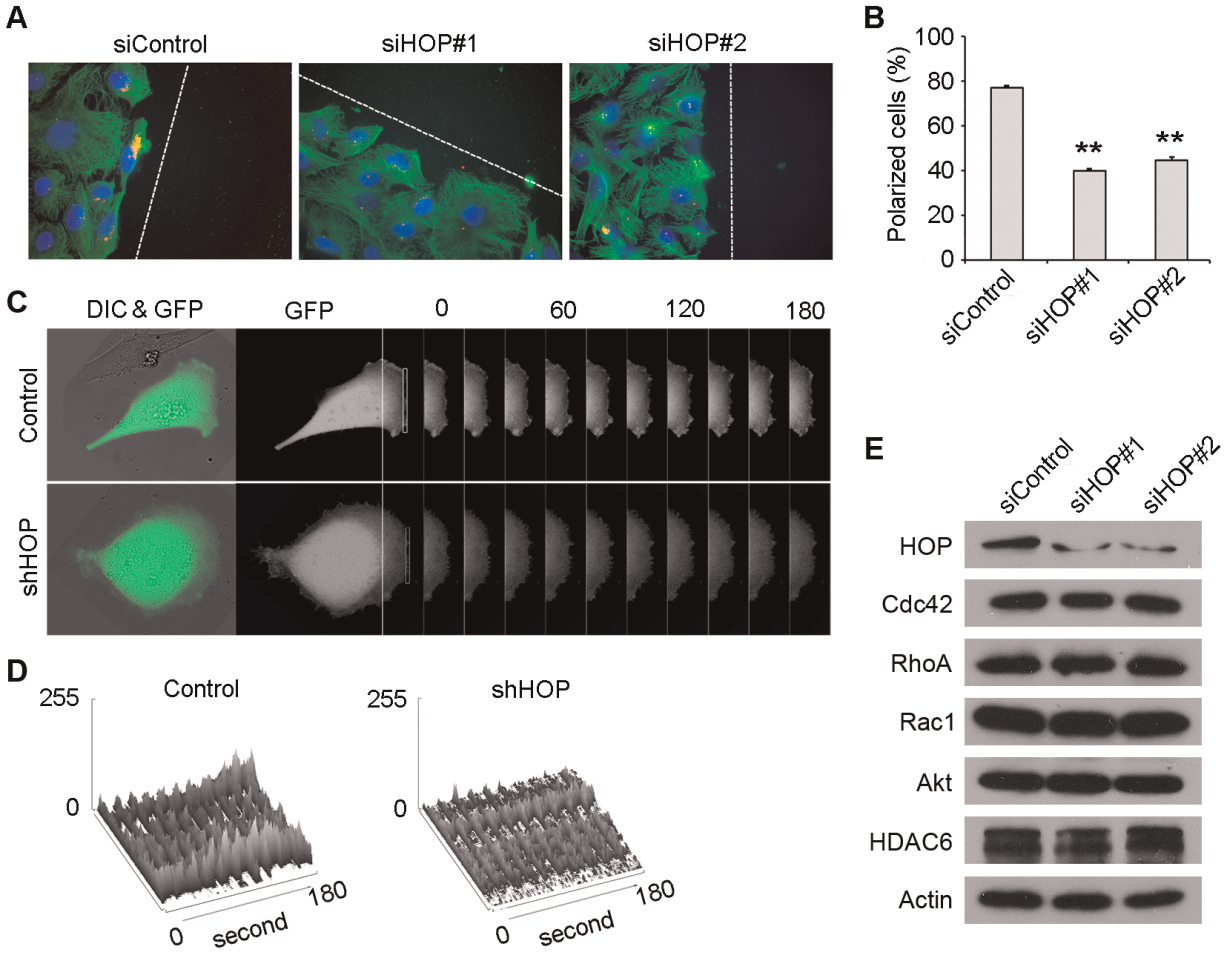
HOP regulates cell polarization and membrane ruffling in migrating cells. **A.** HUVECs transfected with HOP or control siRNAs for 96 hours were scratched, fixed 2 hours later and stained with anti-α-tubulin antibody, anti-γ-tubulin antibody, and DAPI to visualize microtubules (green), centrosomes (red), and nuclei (blue) respectively. Broken white lines indicate the wound directions. **B.** Experiments were performed as in A, and the percentage of polarized cells was quantified. **, p<0.01 versus control. **C.** HUVECs were transfected with GFP along with HOP shRNA or the control vector, and the fluorescence of GFP at the leading edge of cells were recorded at 20-second intervals with the use of the TCS SP5 confocal microscope (Leica). Rectangular regions were selected as indicated to analyze membrane ruffle dynamics. **D.** Experiments were performed as in C, and membrane ruffle dynamics were presented as 3-dimensional surface plots. **E.** Immunoblotting analysis of the expression of several Hsp90 client proteins involved in cell migration and polarity determination in HUVECs transfected with HOP or control siRNAs.

To further investigate the mechanism by which HOP regulates cell migration, we studied the effect of HOP on cell polarization, a critical step of the cell migration process [36], [37]. To examine cell polarization, HUVECs transfected with HOP or control siRNAs were scratched, and fixed 2 hours later for immunofluorescent staining. We defined cells with centrosomes localized between the nuclei and the wound edge as polarized cells. As shown in Figure 4A and 4B, nearly 80% of cells in the control group exhibited polarized characteristics. In contrast, cells transfected with HOP siRNAs showed random centrosome localization.

To better understand the role of HOP in cell polarization, we studied its effect on membrane ruffling at the leading edge, another important feature of migrating cells [38]. HUVECs were transfected with GFP together with HOP shRNA or the control vector, and the fluorescence of GFP at the leading edge of cells was recorded at 20-second intervals to measure membrane ruffle dynamics. We found that HOP shRNA remarkably decreased membrane ruffle dynamics at the leading edge of migrating cells compared with the control vector (Figure 3C and 3D). Thus, HOP plays a critical role in cell polarization and membrane ruffling in migrating cells.

It has been previously shown that in pancreatic cancer cells, the knockdown of HOP expression could decrease the expression levels of several Hsp90 client proteins, including HER2, Bcr-Abl, C-MET, and v-Src [39]. We thus studied whether HOP depletion in HUVECs reduces the levels of known Hsp90 client proteins involved in cell migration and polarity determination, such as Cdc42, RhoA, Rac1, Akt, and HDAC6 [40]–[43]. By immunoblotting, we found that the downregulation of HOP in HUVECs did not affect the expression levels of these proteins (Figure 4E).

### HOP Colocalizes with Microtubules and Interacts with Tubulin

**Figure 5:**
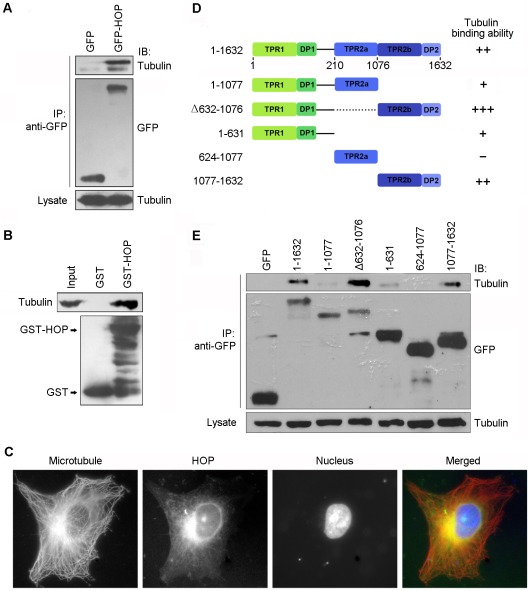
HOP colocalizes with microtubules and interacts with tubulin. **A.** 293T cells were transfected with GFP or GFP-HOP. Anti-GFP immunoprecipitation (IP) assay was performed to examine the interaction between HOP and tubulin. **B.** Purified tubulin was incubated with bacterially purified GST or GST-HOP immobilized on agarose. The presence of tubulin in the GST-pulldown preparation was examined by immunoblotting. **C.** Immunofluorescent images of HUVECs stained with anti-α-tubulin antibody, anti-HOP antibody, and DAPI to visualize microtubules (red), HOP (green), and nuclei (blue), respectively. **D.** A scheme summarizing the interactions of various forms of HOP with tubulin. **E.** 293T cells were transfected with GFP or various truncated forms of GFP-HOP. Cell lysates and anti-GFP immunoprecipitates were immunoblotted with anti-GFP and anti-α-tubulin antibodies.

Microtubules and microtubule-binding proteins play important roles in cell migration and polarization [44]. To test whether HOP functions in cell migration and polarization through interacting with microtubules, we performed immunoprecipitation and GST-pulldown assays. We found that cellular GFP-HOP could coprecipitate with cellular tubulin (Figure 5A). In addition, in the purified system, GST-HOP protein could pulldown tubulin (Figure 5B). We also studied the localization of HOP in HUVECs by immunofluorescent staining. As shown in Figure 5C, HOP could partially colocalize with microtubules in HUVECs. These results thus reveal that HOP interacts with both tubulin and microtubules.

To identify the domains of HOP mediating its interaction with tubulin/microtubules, we constructed a series of plasmids that express various truncated forms of HOP. By immunoprecipitation assay, we found that the HOP mutant lacking the TPR2a domain had the strongest binding affinity to tubulin/microtubules, whereas the TPR2a domain did not interact with tubulin/microtubules (Figure 5D and 5E).

### Addition of Anti-HOP Antibody or Recombinant HOP to the Culture Medium Does Not Affect Vascular Endothelial Cell Migration or Tube Formation

**Figure 6:**
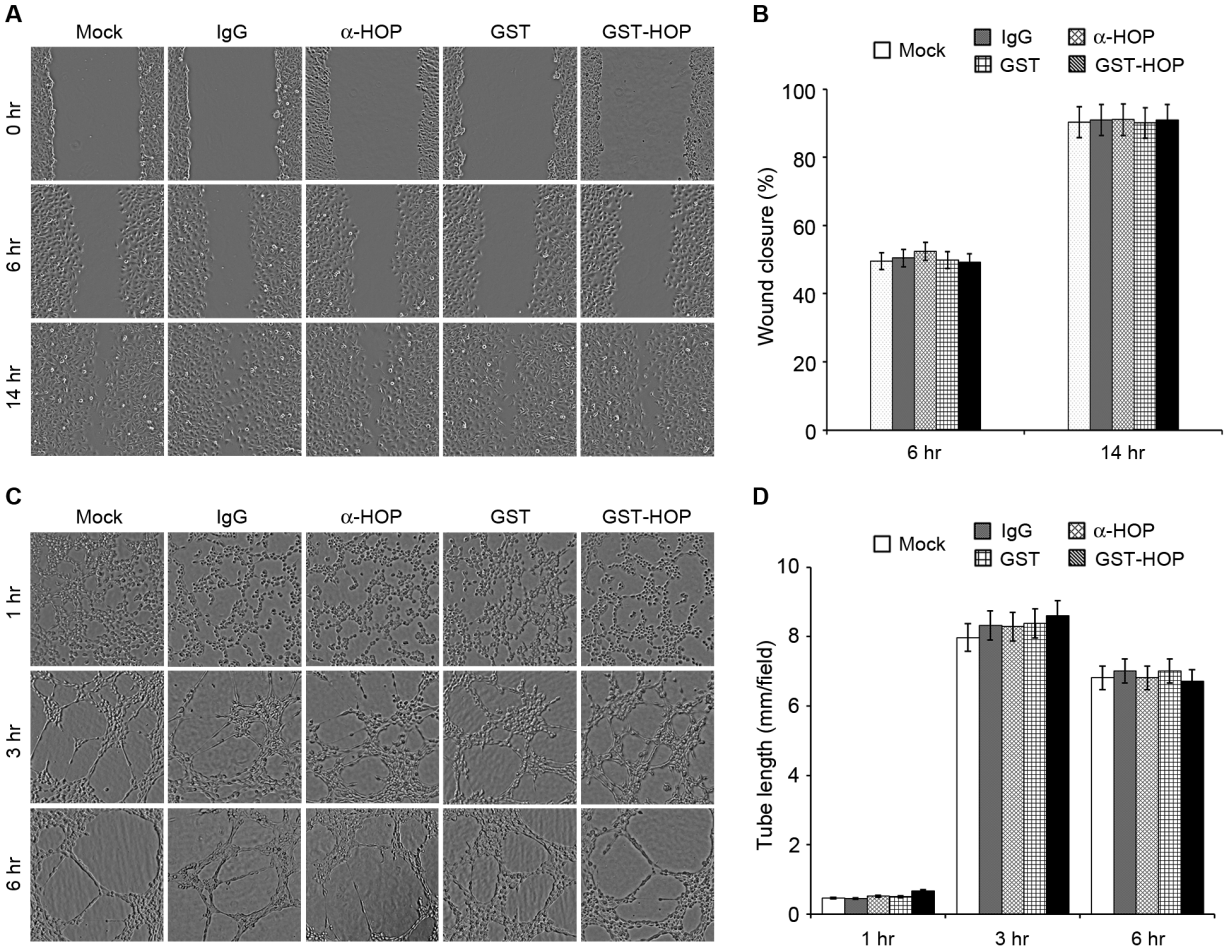
Addition of anti-HOP antibody or recombinant HOP to the culture medium does not affect vascular endothelial cell migration or tube formation. **A.** Scratch wound assays were performed using HUVECs with the addition of anti-HOP antibody, control IgG antibody, purified GST, or purified GST-HOP in the culture medium, and wound margins were imaged 0, 6, or 14 hours later. **B.** Experiments were performed as in A, and the extent of wound closure was quantified. **C.** Tube formation assays were performed using HUVECs with the addition of anti-HOP antibody, control IgG antibody, purified GST, or purified GST-HOP in the culture medium, and photographs were taken 1, 3, and 6 hours later. **D.** Experiments were performed as in C, and the cumulative tube length was measured.

It has been recently reported that Hsp90 and HOP could be released from tumor cells and that extracellular Hsp90 and HOP could promote cell migration and migration [39], [45], [46]. We therefore examined whether HOP could also act also extracellularly in enhancing cell migration. To test this possibility, scratch wound assays were performed by adding anti-HOP antibody, control IgG antibody, purified GST, or purified GST-HOP to the culture medium. As shown in Figure 6A and 6B, the addition of anti-HOP antibody or recombinant HOP to the culture medium does not affect the migration of HUVECs. In addition, we found that the addition of anti-HOP antibody or recombinant HOP to the culture medium does not alter the ability of HUVECs to form tubes (Figure 6C and 6D). These results suggest that extracellular HOP might play a minor role, if there is any, in the migration of vascular endothelial cells.

## Discussion

Angiogenesis, generation of new blood vessels from pre-existing ones, is required for many physiological or pathological processes. In embryo development, angiogenesis is important for providing nutrients and oxygen. During adulthood, angiogenesis occurs in response to some physiological stimuli [47], [48], such as hypoxia and inflammation, for wound healing and repair. Angiogenesis also plays an important role in tumor growth, progression, and metastasis [24], [25]. Thus, it is important to understand how the angiogenic process is precisely controlled. In the present study, by tube formation and capillary sprouting assays, we have identified HOP, a co-chaperone protein, as a novel regulator of angiogenesis. In addition, we have investigated the underlying mechanism and found that HOP regulates angiogenesis by promoting endothelial cell migration.

Several studies have shown that the microtubule cytoskeleton and associated proteins are involved in cell migration and polarization [44]. For example the deubiquitinase CYLD has been demonstrated to regulate angiogenesis by regulating microtubule dynamics and mediating endothelial cell migration [49]. In this scenario, it is tempting to speculate that HOP may function in angiogenesis in a microtubule-dependent manner; however, additional studies are warranted to investigate this possibility. In this study, we have found that HOP colocalizes with microtubules in cells and shown that HOP could interact with tubulin both in cells and in vitro. At present, it remains unclear how HOP and microtubules collaborate to promote cell migration and angiogenesis. It was reported previously that the TPR motif of HOP could form scaffolds to help formation of the chaperone complex [50]. Thus, it is possible that microtubules may function together with HOP to form scaffolds in promoting cell migration and angiogenesis. It is also possible that microtubules may mediate the trafficking of HOP and promote its redistribution in the cytoplasm to facilitate cell polarization and migration.

Structural studies reveal that the interaction between HOP and its target chaperones, Hsp70 and Hsp90, mainly depends on the EEVD motif of the chaperone peptides and the aspartate residue in TPR domains to form a two-carboxylate clamp [6]. In the present study, we have sought to investigate how HOP binds to microtubules/tubulin. Our data show that the domains mediating HOP interaction with microtubules/tubulin may include both TPR1 and TPR2b; however, there is no EEVD motif in tubulin. Considering that TPR domains are generally flexible in the interaction with partner proteins and may have multiple binding modes, different contact interface and different residues might be involved in the interaction of HOP with microtubules/tubulin.

In summary, in the present study we have established a relationship between the HOP protein and angiogenesis by a series of experiments. It will be important to further study the role of HOP in angiogenesis using in vivo models. In addition, the anti-angiogenesis strategy has been employed in cancer treatment [19]–[21]. Given the critical role of HOP in angiogenesis [51], it will be interesting to investigate in the future the potential of HOP as an anti-cancer drug target.
